# Supplementary figures and images for: The classification of movement intention through machine learning models: the identification of significant time-domain EMG features
Source: PeerJ Comput Sci. 2021 Feb 25;7:e379. doi: 10.7717/peerj-cs.379 (PMC7959624; doi:10.7717/peerj-cs.379)

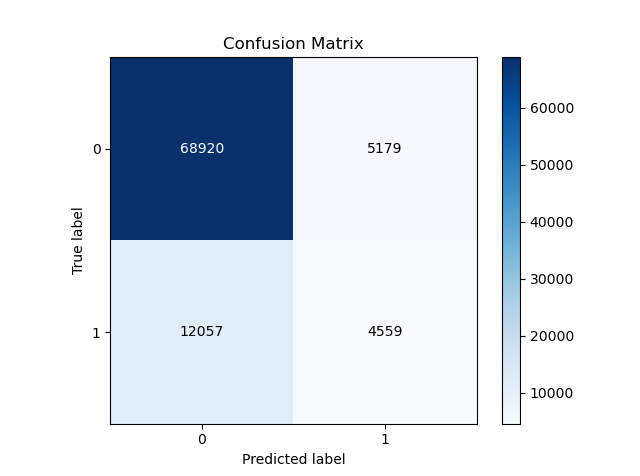

Supplement: Supplemental Information 2 [file peerj-cs-07-379-s002.zip › knn/all.png]

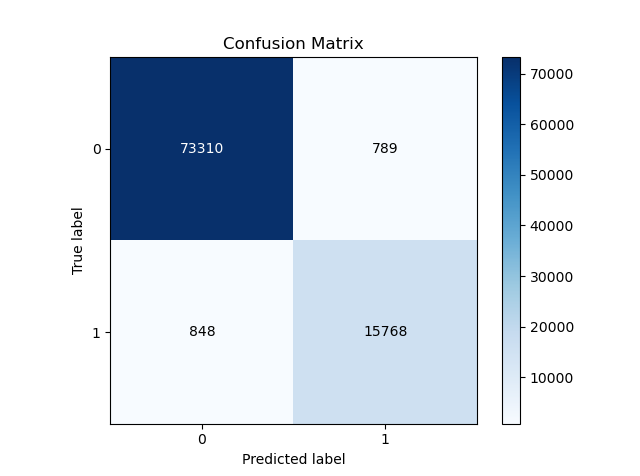

Supplement: Supplemental Information 2 [file peerj-cs-07-379-s002.zip › knn/best_feat.png]

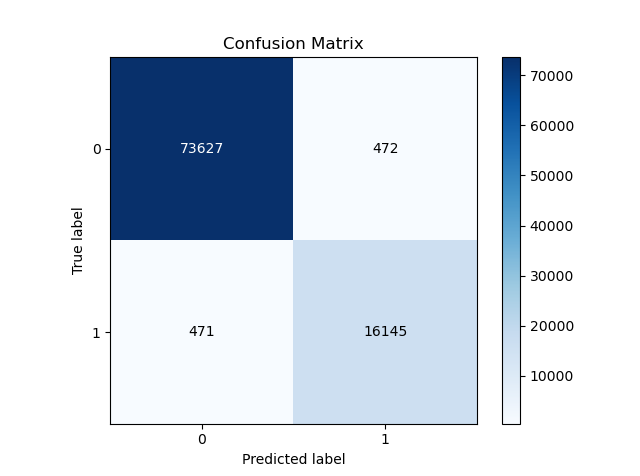

Supplement: Supplemental Information 2 [file peerj-cs-07-379-s002.zip › knn/opt_.png]
